# Structural basis of RNA cap modification by SARS-CoV-2

**DOI:** 10.1038/s41467-020-17496-8

**Published:** 2020-07-24

**Authors:** Thiruselvam Viswanathan, Shailee Arya, Siu-Hong Chan, Shan Qi, Nan Dai, Anurag Misra, Jun-Gyu Park, Fatai Oladunni, Dmytro Kovalskyy, Robert A. Hromas, Luis Martinez-Sobrido, Yogesh K. Gupta

**Affiliations:** 1 Greehey Children’s Cancer Research Institute, University of Texas Health at San Antonio, 8403 Floyd Curl Drive, San Antonio, TX 78229 USA; 2 Department of Biochemistry and Structural Biology, University of Texas Health at San Antonio, 7703 Floyd Curl Drive, San Antonio, TX 78229 USA; 3 New England Biolabs, 240 County Road, Ipswich, MA 01938 USA; 4 Texas Biomedical Research Institute, San Antonio, TX 78227 USA; 5 Division of Hematology and Oncology, Department of Medicine, University of Texas Health at San Antonio, 7703 Floyd Curl Drive, San Antonio, TX 78229 USA

**Keywords:** Holoenzymes, SARS-CoV-2, X-ray crystallography

## Abstract

The severe acute respiratory syndrome coronavirus-2 (SARS-CoV-2), the causative agent of COVID-19 illness, has caused millions of infections worldwide. In SARS coronaviruses, the non-structural protein 16 (nsp16), in conjunction with nsp10, methylates the 5′-end of virally encoded mRNAs to mimic cellular mRNAs, thus protecting the virus from host innate immune restriction. We report here the high-resolution structure of a ternary complex of SARS-CoV-2 nsp16 and nsp10 in the presence of cognate RNA substrate analogue and methyl donor, S-adenosyl methionine (SAM). The nsp16/nsp10 heterodimer is captured in the act of 2′-O methylation of the ribose sugar of the first nucleotide of SARS-CoV-2 mRNA. We observe large conformational changes associated with substrate binding as the enzyme transitions from a binary to a ternary state. This induced fit model provides mechanistic insights into the 2′-O methylation of the viral mRNA cap. We also discover a distant (25 Å) ligand-binding site unique to SARS-CoV-2, which can alternatively be targeted, in addition to RNA cap and SAM pockets, for antiviral development.

## Abstract (web summary)

Specific non-structural proteins (nsp) of SARS coronaviruses are involved in methylation of virally encoded mRNAs to mimic cellular mRNAs for protection against host innate immune restriction. Here, the authors present a high resolution structure of SARS-CoV-2 nsp16/nsp10 ternary complex in the presence of cognate RNA substrate analogue and methyl donor, S-adenosyl methionine, revealing unique ligand-binding sites that may represent alternative targets for antiviral development.

## Introduction

The massive global pandemic with high morbidity and mortality makes SARS-CoV-2 one of the deadliest viruses in recent history^1^. To develop effective therapies, we need a better understanding of the mechanisms that permit the virus to invade cells and evade host immune restriction. SARS-CoV-2 is an enveloped β-coronavirus with a large, complex positive-sense single-stranded RNA genome^2^. To hijack the host translation machinery for propagation, enzymes encoded by the genome of coronaviruses (CoVs) modify the 5′-end of virally encoded mRNAs by creating a cap^3^. RNA capping in CoVs involves activities of several nonstructural proteins (nsps): nsp13, a bifunctional RNA/NTP triphosphatase (TPase) and helicase; nsp14, a bifunctional 3′→5′ mismatch exonuclease, and mRNA cap guanine-N7 methyltransferase; nsp16, a cap ribose 2′-O methyltransferase; and an elusive guanylyl transferase^4–7^.

Nsp16 forms an obligatory complex with nsp10 to efficiently convert client mRNA species from the Cap-0 (^me7^G_o_pppA_1_) to the Cap-1 form (^me7^G_o_pppA_1m_), by methylating the ribose 2′-O of the first nucleotide (usually adenosine in CoVs) of the nascent mRNA using SAM (S-adenosyl methionine) as the methyl donor^4,8^. While both Cap-0 and Cap-1 forms promote the recruitment of the host translation factor eIF4E, the Cap-0 form favors the binding of interferon-induced proteins with tetratricopeptide repeats 1 (IFIT1) to viral RNAs^9^. Thus, the Cap-1 form not only enhances translation, but also serves to avoid induction of the innate immune response mediated by interferon stimulated genes such as IFIT^10–12^. Genetic disruption of SARS-CoV *nsp16* markedly reduces (10-fold) the synthesis of viral RNA^13^. Hence, the ablation of nsp16 activity should trigger an immune response to CoV infection and limit pathogenesis^10,11^. It has been shown that live vaccination with nsp16-defective SARS-CoV-1 or an immunogenic disruption of the nsp16-nsp10 interface protects mice from an otherwise lethal challenge^14,15^, making nsp16/nsp10 an attractive drug target.

Crystal structures of SARS-CoV-1 nsp16/nsp10 in complex with SAM/SAH or sinefungin, but without an RNA cap, have been reported^4,8,16^, so that key information about the catalytic mechanism of mRNA capping in CoVs, and SARS-CoV-2 in particular, is still missing. To understand the determinants of RNA cap modification and help guide the development of SARS-CoV-2 antiviral therapies, we have now succeeded in solving the high-resolution structure (to 1.8 Å resolution) of SARS-CoV-2 nsp16/nsp10 in complex with the methyl donor (SAM) and its target, RNA cap ^me7^G_o_pppA_1_.

We show how this enzyme is specifically adapted to bind and methylate the RNA cap. The structure provides a snapshot of pre-catalytic state of methyl transfer from SAM to 2′-OH of ribose of the first transcribing nucleotide of the mRNA cap. It also reveals the nature of conformational change in nsp16, the catalytic subunit of nsp16/nsp10 complex, induced by RNA cap binding. Another striking finding includes an alternative ligand-binding site in nsp16 with distinct capability to accommodate small molecule ligands. Finally, we map the acquired mutations in SARS-CoV-2 nsp16. One of these mutation hotspots showed high frequency in COVID-19 strains associated with New York City outbreak. Together, our work provides a solid framework from which therapeutic modalities may be designed by targeting different ligand-binding sites of nsp16, including RNA cap and SAM pockets, for the treatment of COVID-19 and emerging coronavirus illnesses.

## Results and discussion

### Crystallization

**Fig. 1:**
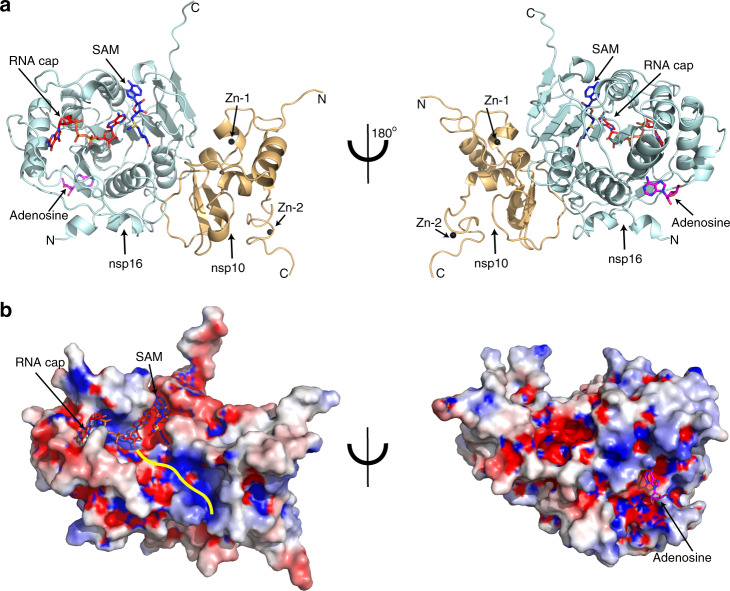
Overall structure of the SARS-CoV-2 nsp16/nsp10 ternary complex. **a** Subunit arrangement of nsp16 (cyan) and nsp10 (orange cartoons) proteins with respect to RNA cap (red stick), and S-adenosyl methionine or SAM (blue stick). Proteins and nucleotides are shown in cartoon and stick modes, respectively. Black spheres, Zn^2+^ atoms bound to nsp10; Magenta stick, adenosine bound to nsp16. **b** Electrostatic surface representation of nsp16/nsp10 with saturated blue and red at +5 kT/e and −5 kT/e, respectively. A yellow line represents a tentative path for downstream RNA sequence calculated by superposing the target bases in current, VP39, and Dengue NS5 ternary complexes.

**Fig. 2:**
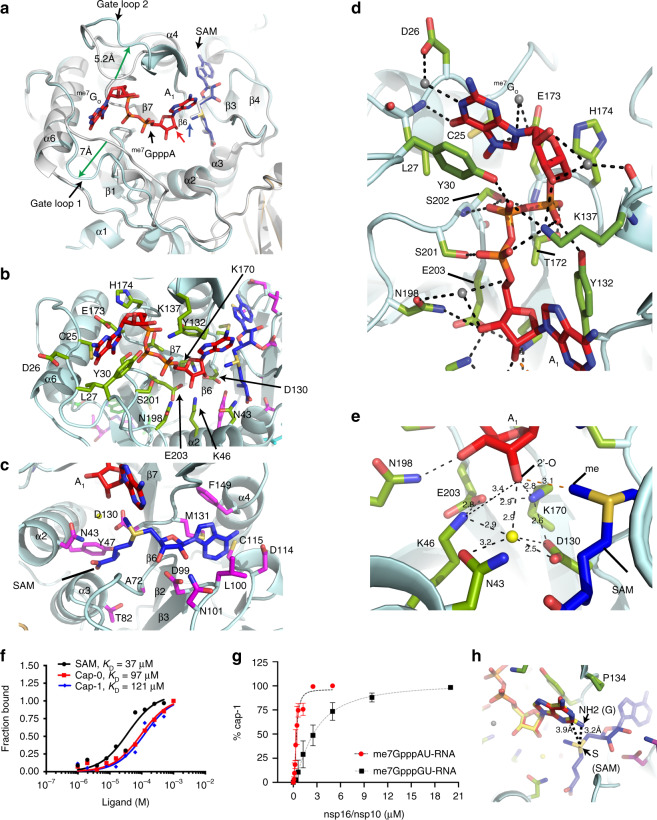
Binding modes of RNA cap analogues and SAM and mechanism of methyl transfer. **a** Overlay of a binary (S-adenosyl methionine or SAM-bound; gray cartoons) and ternary (SAM, RNA cap-bound; light cyan cartoons) complexes shows outward motions (green arrows: 7 Å in gate loop 1, and 5.2 Å in gate loop 2) after RNA cap binding to nsp16. RNA cap, red stick; SAM in ternary complex, blue stick; SAM in binary complex, gray stick. **b** green sticks; nsp16 residues that interact with RNA cap. **c** magenta sticks; nsp16 residues that contact with SAM. **d** A close-up view of Cap-nsp16 interactions reveals a network of hydrogen bonding with successive phosphates, ^me7^G_O_ and A_1_ nucleotides of cap. Water, gray spheres, h-bonds, black dashed lines. **e** A water (yellow sphere) coordinates with the target 2′-O atom of A_1_, and catalytic tetrad residues and N43. The methyl group of SAM is positioned for direct in-line attack from the 2′-O. **f** Binding isotherms and fitting of data for nsp16 binding to RNA cap-0 (^me7^GpppA), cap-1 (^me7^GpppAm) analogues, and SAM. Each data point represents average of two independent experiments (*n* = 2). **g** The 2′-O methyltransferase activity measured as percentage of Cap-0 to Cap-1 conversion is plotted against nsp16/nsp10 protein concentration. Higher enzymatic activity is observed on an RNA substrate with A (red circles) as the target base for 2′-O methylation (N_1_), compared to an identical RNA but with G (black square) as N_1_ or initiating nucleotide. Results are average of three independent experiments (*n* = 3) with one standard deviation (s.d.) for each RNA shown as error bars. Source data are provided as a Source Data file. **h** Guanine base (yellow stick) is modeled at N_1_ position of cognate adenine (red stick). The N2 amine of guanine intrudes into the SAM pocket and may be repelled by positively charged sulfur of SAM (blue stick).

We co-purified full-length SARS-CoV-2 nsp16 and nsp10 proteins as a complex from *E. coli*, mixed with nucleoside drugs such as adenosine or 5′-methylthioadenosine, and conducted crystallization screenings (see Methods for details). We anticipated that these drugs may occupy the binding sites of SAM or RNA cap due to their common purine ring plus ribose. Since nucleoside analogues exhibit antiviral activity^17–19^, an nsp16/nsp10/adenosine complex could then serve as a starting point for medicinal chemistry to generate more effective drug candidates. Next, we soaked these crystals with ^me7^G_o_pppA_1_ representing the Cap-0 state of the RNA cap and substrate of this complex. We determined the ternary structure of nsp16/nsp10 by molecular replacement using the SARS-CoV-1/SAM binary complex (PDB ID 3R24)^8^ structure as a template (Supplementary Table 1). The initial difference maps indicated electron densities for SAM and ^me7^G_o_ppp**A**_**1**_ cap in their respective binding pockets. Even though SAM was not included in co-crystallization or soaking experiments, its electron density was unambiguously identified in the difference maps (see methods section, and Figs. 1 and 2, Supplementary Fig. 2a–d). This observation is consistent with the copurification tendency of SAM or SAH during isolation from expression hosts, as seen previously with NS5 of ZIKA and VP39 of vaccinia virus cap 2′-O MTases^20,21^. Unexpectedly, we observed electron density on the opposite face of nsp16, >25 Å away from the catalytic pocket, that was consistent with adenosine, that we had included in our crystallization mixture (Fig. 1, Supplementary Figs. 2 and 3). Further examination showed that this alternative ligand-binding pocket was partially composed of amino-acid residues unique to SARS-CoV-2 (Supplementary Fig. 1).

### Overall structure

Nsp16 adopts a canonical S-adenosyl methionine (SAM)-dependent methyltransferase (SAM-MTase) protein fold^22^, with slight variations^8,16^. Its protein sequence displays a sequential order of secondary structural elements: ·β1α1α2β2α3β3β4·β5β6·α4β7α5β8·β9α6·β10·α7β11β12, wherein the ‘·’ denotes a 3_10_-helix (Fig. 1, Supplementary Fig. 1). The nsp16 MTase fold consists of a centrally located twisted β sheet of eight strands flanked by two alpha-helices on one side and three helices on the other. The β sheet displays a continuum of four antiparallel (β1β8β9β7) and four parallel (β6β2β3β4) β strands, with the cap analogue substrate situated within the confluence of these two halves. Loops emanating from strands β9, β7, and β6 form a deep groove in the center to accommodate the RNA cap, whereas the methyl donor SAM is bound within a cavity formed by the loops originating from strands β6 and β2. The protein chain emerging from helix α4 runs across this groove and folds into a subdomain (·β10·α7β11β12) that stabilizes the bottom portion of nsp16 (Fig. 2). The putative adenosine binding pocket we identified is located ~25 Å away at the back of the catalytic pocket (Fig. 1).

Nsp10 is a 139 amino-acid long zinc-binding protein that stimulates the enzymatic activity of nsp16^4,8,16^. We traced all functional regions known for protein–protein and protein–metal binding in nsp10, with the exception of the N-terminal 17 residues that appear to be disordered in our structure, but form an α-helix in the binary state (CoV-1 nsp10) in the absence of a cap structure^4,8,16^. The N-terminus of this helix runs in the direction of the cap-binding site of nsp16 but locates >20 Å away from the catalytic center. Nsp10 adopts a structural fold with two distinct Zn-binding modules, including a gag-knuckle-like fold^23^. Binding of the RNA cap did not induce any major conformational change in nsp10.

### RNA cap analogue binding

We compared our ternary structure with bound substrate to that of SARS-CoV-1 nsp16/nsp10 bound to SAM (PDB ID 3R24), but without substrate. While the cores of the nsp16 and nsp10 proteins remain largely unperturbed (with an RMSD of 1.11 Å for 292 Cα atoms between the prior CoV-1 structure and our own), we found significant deviations in two regions of nsp16 that constitute the substrate-binding pocket. We refer to these regions as gate loop 1 (amino acids 20–40) and gate loop 2 (amino acids 133–143) (Fig. 2). The binding of the Cap-0 substrate results in an ~180° outward rotation of gate loops 1 and 2 from their positions in the binary structure displacing them by 7 Å and 5.2 Å, respectively. The resultant widening of the pocket allows accommodation of the RNA cap substrate, and engages the *N*^7^-methyl guanosine base of Cap-0 (^me7^G_o_) in a deep groove formed by residues of gate loop 1 on one side and gate loop 2 on the other (Fig. 2a, b, d, f, Supplementary Movie 1, Supplementary Fig. 4). The loop region immediately downstream of Pro134 that is part of gate loop 2 also flips ~180° in the ternary complex to accommodate the A_1_ nucleotide of the cap (Fig. 2a). Gln28 and Asn29 (gate loop 1) orient outward, whereas the side chain of invariant Tyr30 rotates inward, thereby forming a cleft to stabilize the purine ring of ^me7^G_o_ through a π-π stacking interaction (Fig. 2d).

The opposite face of the ^me7^G_o_ purine ring is stabilized by a H-bond between an oxygen of Glu173 and the positively charged N7 of ^me7^G_o_. The methyl group of ^me7^G_o_ is stabilized by hydrophobic interactions with Cys25 (gate loop 1) and Ser 202 from the loop connecting the β8 and β9 strands (Fig. 2b, d). These specific interactions with the positively charged N7 and the methyl moiety of the ^me7^G_o_ may confer substrate selectivity against the uncharged G_o_pppA^4^. In gate loop 2, Asn138 rotates outward and the side chain of the invariant Lys137 rotates ~180° inward and forms stabilizing electrostatic interactions with all 3 phosphates of the Cap-0 structure and the 3′-O of the ^me7^G_o_ ribose. Tyr30 and Lys137 form a partial enclosure to restrict the movement of the terminal residue (^me7^G_o_) of the RNA cap (Fig. 2b, d, Supplementary Movie 1). Gate loop 1 runs perpendicular to the purine ring of ^me7^G_o_ such that the backbone amide of Leu27 forms a hydrogen bond with O6 of ^me7^G_o_ to confer specificity for guanine. The ribose sugar of ^me7^G_o_ is sandwiched by the sidechains of Lys137 and His174 in gate loop 2 (Fig. 2b, d). The three phosphates of ^me7^G_o_pppA_1_ are stabilized through H-bonds with residues located in the loop regions emanating from the β6 (Tyr132, Lys137), β7 (Thr172 and His174), and β8 (Ser 201 and Ser 202) strands (Fig. 2d, Supplementary Fig. 4). By comparing our structure with those for the 2′O MTases of Dengue (PDB ID: 5DT0) and Vaccinia (PDB ID: 1AV6) viruses, we note that only one aromatic residue (Tyr30 in SARS-CoV-2 nsp16 and Phe25 in dengue virus NS5) makes the stacking interaction with the ^me7^G_o_ of the RNA cap. In contrast, two aromatic residues (Tyr22, Phe180) intercalate the ^me7^G_o_ of RNA cap in VP39 of vaccinia virus (PDB ID: 1AV6). Also, the distance between N_1_ and ^me7^G_o_ bases remains unchanged (~10 Å), and the SAM/SAH and N_1_ in all three structures overlay well in three structures. However, the terminal ^me7^G_o_ base of CoV-2 RNA cap rotates ~180° around the γ-phosphate (compared to Dengue virus) to assume a radically different orientation, suggesting a diverse geometric arrangement of the RNA cap in RNA/DNA viruses (Supplementary Fig. 2e). This distinct feature of RNA cap can be exploited to design selective antivirals for SARS-CoV-2.

### Methyl donor (SAM) binding

SAM occupies the same pocket, and orients similarly as in the binary complex (PDB ID 3R24)^8^. Several loops emanating from the C-terminal ends of the four parallel β strands (β2, β3, β4, and β6) of the central β sheet form a deep groove to accommodate SAM through an extensive network of electrostatic, hydrophobic, and van der Waals interactions (Fig. 2c, f). In contrast to the substrate-binding pocket, which is largely basic, the SAM binding pocket in nsp16 is enriched by negatively charged residues (Fig. 1b). The sidechains of Asp99 and Asn101 from strand β3 form hydrogen bonds with terminal oxygens of the ribose sugar, whereas the purine ring of SAM is partially circled by sidechains of Leu100 (β3) Asp114, Cys115 (β4), and Met131 (β6), and Phe149. Asp114 makes a base-specific interaction with SAM via a hydrogen bond with the N6 amino group of the purine ring. A four amino-acid long stretch (Met131 to Pro134) that corresponds to gate loop 2 intrudes into the SAM and cap-binding pockets and separates the two purine rings of SAM and A_1_ (the target adenine) by ~10 Å. This separation favorably orients the ribose sugar of A_1_ and the donor methyl group of SAM for 2′-O methylation. The carboxy end of SAM is stabilized by charged interactions from the sidechains of Asn43 and Tyr47 and the backbone amide of Gly81 (Fig. 2c, Supplementary Fig. 4).

### Methylation of target adenine of RNA cap

Based on the crystal structures of SAM and SAH-bound SARS-CoV-1 nsp16/nsp10 complexes, a catalytic mechanism that involves a tetrad consisting of the invariant Lys46, Asp130, Lys170, and Glu203 has been proposed^8,16^. Our ternary complex has allowed us to examine the model in the presence of the Cap-0 substrate. The catalytic pocket adopts a conical shape where residues of the catalytic tetrad circle a water molecule at the bottom of the cone, whereas the target atom (2′-OH) of the A_1_ nucleotide resides at the tip of the cone. The methyl group of SAM is positioned 3 Å from the 2′-OH of A_1_, available for a direct in-line attack from the 2′-OH (Fig. 2c, e). An earlier study posits that the target 2′-OH of the ribose in a ternary complex would initially occupy the position of the water while Lys46 would become a deprotonated general base and activate the 2′-OH to attack the methyl group of SAM^16^ with Asp130 stabilizing the positive charge of the methyl group^16^. However, our structure shows that this water molecule, located 3 Å from the target 2′-OH of the A_1_ base ribose, remains unperturbed in the ternary structure of CoV-2 and binary structures of CoV-1^8,16^ nsp16. If the ribose were to occupy the position of water as suggested^16^, the adenine ring of A_1_ would sterically clash with SAM. Also, Lys170 is much closer to the 2′OH (2.8 Å) than Lys46 (3.4 Å) and is held in place by charged interactions from the sidechains of Asp130 and Glu203 from both sides (Fig. 2e, Supplementary Fig. 4). Thus, in our ternary complex structure, Lys170 best matches the one activating the 2′-OH in other viral 2′-O MTases (e.g., Lys180 of dengue virus NS5^24^, and Lys175 of vaccinia virus VP39^25^), and likely serves as a general base during 2′-O methyl transfer in SARS-CoV-2. One possible role of the water molecule is that it cooperates with Asp130 to transiently stabilize the transfer of the positively charged methyl group from SAM to the 2′-O of A_1_ base. We propose that once occupied by SAM, the catalytic pocket is primed for A_1_ base binding and catalysis. This may be a general mechanism for 2′-O methylation for all coronaviruses.

Supporting this concept, nsp16/nsp10 showed robust 2′-O methyltransferase activity. Consistent with its specificity in vivo, we observed a marked reduction in activity when the first transcribing nucleotide (N_1_) was changed from the cognate adenine to non-cognate base guanine (Fig. 2g). While this has been observed previously^8^, the structural basis for base discrimination has not been defined. While we see no base-specific contact for the N_1_ base in our ternary structure, modeling of other bases (G/C/U) at this position provides some clues as to how specificity for A arises. The Pro134 resides at van der Waals distance from the purine ring of the N_1_ base. A guanine at N_1_ will not alter this interaction but the N2 amino group of guanine intrudes into the SAM pocket thereby reducing the distance between guanine and SAM by ~0.7 Å. As a result, the positively charged sulfur of SAM may repel the purine ring of guanine leading to reorientation of its target sugar 2′-OH to a position unfavorable for methyl transfer. A pyrimidine base (U/C) at N_1_ position will sterically clash with the side chain of Tyr132 (Fig. 2h, Supplementary Fig. 2f). Although all components required for methylation are present in our crystals, we have not observed methylation of 2′-OH, possibly due to lower abundance of methyl and sulfur groups of SAM (75% occupancy) and requirement for additional RNA sequence downstream of the target A_1_ base^8^. Thus, the ternary structure that we have described may represent a pre-reaction state of 2′-O methyl transfer.

### Acquired mutations in SARS-CoV-2 and their implications

To better understand the role of acquired mutations in SARS-CoV-2 nsp16 (compared to CoV-1), we aligned nsp16 sequences from nine coronavirus strains representing all three sub-classes (α, β, and γ), and mapped their positions in the current structure (Supplementary Fig. 1). Although most residues that participate in catalysis and substrate/SAM binding are conserved across species, notable differences were found (Supplementary Figs. 1, 3b). Compared to CoV-1, acquired mutations span the entire nsp16 sequence: E32D and N33S (gate loop 1); K135R (gate loop 2); and S188A, A209C, T223V, N265G, Y272L, E276S, V290I, and I294V (Supplementary Figs. 1, 3b). Given the role of gate loop regions in RNA substrate binding, mutations E32D and N33S in gate loop 1 and K135R in gate loop 2 may influence RNA binding or enzyme kinetics. Interestingly, a recent transcriptomic profiling study of SARS-CoV-2 strains associated with the New York City outbreak found high occurrence of a mutation (20755:A > C) that will change serine 33 to an arginine in gate loop 1 of SARS-CoV-2 nsp16^26^. Future studies will reveal the impact of this mutation on enzyme activity.

**Fig. 3:**
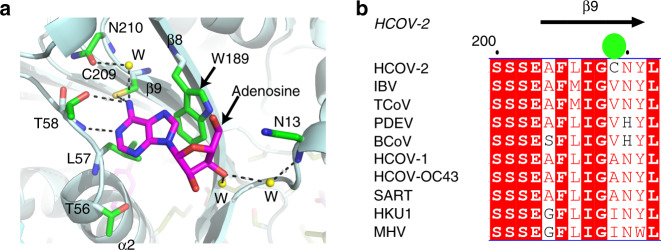
Alternative ligand-binding site in nsp16. **a** nsp16 residues that participate in adenosine (magenta stick) binding are shown as green sticks; water molecules are shown as yellow spheres. **b** Cys209 (green sphere) in the β9 strand, which is present in nsp16 of SARS-CoV-2, but no other CoV strain.

Three specific mutations (S188A (β8), A209C (β9), and E276S (α7)) form part of the adenosine binding pocket we have described 25 Å from the substrate-binding site. Two of the mutations, A209C (β9) and E276S (α7), are not present in any other CoV strain, whereas S188A, is present in one human strain (HKU1), two avian strains (TCoV and IBV) and one mouse strain (MHV) (Fig. 3, Supplementary Fig. 3b). The Ala188 and Cys209 in CoV-2 nsp16 reside at one end of the antiparallel β strands β8 and β9, respectively. Since the residues located at the other end of these interconnecting β strands (invariant Ser 201 and Ser 202) recognize the α and β phosphates of the RNA cap, the binding of adenosine (or a larger ligand) to this alternative site may influence the RNA cap binding. Moreover, the α7 helix interconnects the c-terminal extension (amino acids 256–298), MTase core, and the N-terminal domain (α2) of nsp16. The E276 (α7) in CoV-1 interacts with K216 located at the surface of MTase core. This charged interaction pair is broken in CoV-2 due to E276S and K216R mutations. However, binding of a small molecule ligand such as adenosine along with van der Waal interactions between S276 and A188 stabilizes this region of nsp16 in CoV-2.

At ~25 Å away, and on the back of the catalytic center, this ligand-binding pocket does not present obvious characteristics for adenine specificity such as an aspartic acid or asparagine residue, which would usually make a hydrogen bond with the N6 amino group of adenine. Nonetheless, it displays two similarities with the SAM binding pocket: the purine ring in both pockets resides in an acidic environment, and both pockets harbor a cysteine near N6 of the purine ring. In the SAM pocket, Cys115 is 4.1 Å from the N6 amino group, with a ~64° angle between N1 and the C6 atoms of the adenine ring with respect to Cys115 (Fig. 2c). An equivalent cysteine in the adenosine pocket, Cys209, is at 3.5 Å away from, and at a ~85° angle to the N6 of adenine. The purine ring of adenosine is further stabilized by stacking and electrostatic interactions with Trp189 and Thr58, respectively. Its 5′ tail, while exposed to solvent, interacts with Asn13. (Fig. 3, Supplementary Fig. 4). Thus, the lack of extensive interactions for adenosine and its high temperature factors (compared to RNA cap analogue and SAM) suggest that this pocket is probably not specific for adenosine but could accommodate other small molecules with a heterocyclic ring. Supporting this hypothesis, recent crystal structures of SARS-CoV-2 nsp16 showed a sugar moiety (β-D-fructopyranose, PDB ID: 6W4H), or modified nucleotides (^me7^GDP, PDB ID: 6WQ3; ^me7^GTP, PDB ID: 6WRZ) occupying the position of adenosine (Supplementary Fig. 3c–f). In SARS-CoV-1 nsp16 (PDB ID: 2XYR)^16^, a metal ion (Mg^2+^) is present in the vicinity of the adenosine/^me7^GDP/^me7^GTP/β-D-fructopyranose/^me7^GTP ligands binding site (Supplementary Fig. 3e). Thus, we propose that this distant region endows nsp16 with the unique ability to bind small molecules outside of the catalytic pocket.

In conclusion, we have presented here a high-resolution structure of a ternary complex of SARS-CoV-2 RNA cap/nsp16/nsp10 complex captured just prior to ribose 2′-O methylation. The structure also reveals the basis of an ‘induced fit’ model of the RNA cap binding and 2′-O methylation of the first transcribing nucleotide of SARS-CoV-2 genome. Also, we describe a distantly located ligand-binding site in nsp16/10 capable of accommodating small molecules outside of the catalytic pocket, which may be considered, in addition to SAM- and RNA cap-binding pockets, in the development of antiviral therapies to treat SARS-CoV-2 infections.

## Methods

### Protein expression and purification

The coding sequences of nsp16 (NCBI reference sequence YP_009725311.1) and nsp10 (NCBI reference sequence: YP_0009725306.1) of the Wuhan seafood market pneumonia virus isolate Wuhan-Hu-1 (NC_045512) were cloned into a single pETduet-1 vector downstream to a 6XHis tag sequence. This plasmid was transformed into an *E. coli* expression strain BL21 (DE3). The transformed cells were grown in Terrific Broth medium supplemented with ampicillin at 37 °C. Protein expression was induced by adding 0.4 mM isopropyl β-D-thiogalactopyranoside (IPTG) at OD_600_ = 0.6 followed by continued incubation of the cultures for 14 h at 18 °C. Cells were then harvested by centrifugation at 8983 × *g* for 20 min. Thereafter, cells were re-suspended in ice-cold lysis buffer. Cell lysis was accomplished using a microfluidizer (Analytik, UK). The lysate was centrifuged at 158,000 × *g* for 40 min and passed through a 0.22 µm filter. The clarified soluble fraction was loaded on to a Nuvia IMAC column (Bio-Rad) pre-equilibrated in binding buffer containing 25 mM Tris-HCl pH 8.0, 0.2 M NaCl, 0.1 mM TCEP, 5% Glycerol. The proteins were eluted by increasing the concentration of imidazole. The 6XHis tag was then proteolytically removed.

The tag-free sample was re-applied to the IMAC column to separate the un-cleaved protein fraction. The proteins were purified by successive passage through HiTrap heparin and Superdex 75 columns (GE Healthcare). The nsp16/nsp10 complex was eluted as a single homogenous species in a final buffer containing 25 mM Tris-HCl pH 8.0, 0.5 M NaCl, 0.1 mM TCEP, 10% glycerol and 5 mM MgSO_4_. The purified protein complex was concentrated to 15 mg/mL, mixed with 5 molar excess of adenosine, and subjected to extensive crystallization trials.

### Crystallization

The nsp16/nsp10 complex was crystallized in the presence S-adenosyl methionine (SAM) and adenosine. Initial crystals were grown by the sitting drop vapor diffusion method. After 3–4 rounds of optimizations by varying pH, precipitant and the salt concentrations, we grew larger crystals amenable to synchrotron radiation. Diffraction-quality crystals were grown in a crystallization solution containing 10% (v/v) MPD, 0.1 M HEPES pH 7.0. We soaked these crystals with ^me7^GpppA RNA analogue representative of Cap-0 structure. Before data collection, the cocrystals were cryoprotected by serial soaks in a solution containing original mother liquor and increasing concentrations (0–20% v/v) of ethylene glycol, and then flash-frozen in liquid nitrogen.

### X-ray diffraction data collection and structure determination

Crystals of the nsp16/nsp10/SAM/Cap-O/adenosine complex diffracted X-rays to 1.8 Å resolution with synchrotron radiation. The crystals belong to space group P3_1_21 with unit cell dimensions a = b = 168 Å, c = 52.3 Å, c = 313.9 Å, α = β = 90°, and γ = 120°, and with one nsp16/nsp10 heterodimer per asymmetric unit. All data (measured at wavelength 0.9792 Å) were indexed, integrated, and scaled using XDS^27^, aimless, and various ccp4 suite programs (truncate, freeflag, and mtz2various)^28^. The structure was solved by molecular replacement using a binary complex (SAM-bound) of nsp16/nsp10 (PDB ID: 3R24)^8^ structure as a template in Phaser^28^. The resulting maps indicated the electron densities for SAM, RNA Cap-0 analogue, and adenosine, and regions in nsp16 with extreme conformational changes (gate loops 1 and 2). To confirm the identity of bound cofactor (SAM) or its reaction by-product S-adenosyl homocysteine (SAH) in the SAM binding pocket, we calculated the unbiased Fo–Fc maps by excluding ligands from the refinement and phase calculations. We refined the model by including SAH or SAM or SAM with the methyl group modeled in the opposite direction from the target 2′-OH. By comparing these results, we concluded that SAM predominantly occupied this site with methyl group pointing in the direction of the target 2′-OH. As presented, the SAM was refined with 100% occupancy except for the sulfur and methyl moieties, which were refined at 75% occupancy (Supplementary Fig. 2a–d). We iteratively rebuilt and refined the model using the programs Coot^29^ and REFMAC^28^. The final model was refined to 1.8 Å resolution with R_free_ and R_work_ values of ~18.1% and 14.3%, respectively, (Supplementary Table 1). A Ramachandran plot for the final model shows 94.1% of the residues in the most preferred regions, 4.6% in the additionally allowed regions, and 1.2% in the disallowed regions. All figures that depict structural models were generated using Pymol (The PyMOL Molecular Graphics System, Version 2.0 Schrödinger, LLC.).

### Determining affinities for nsp16/nsp10 ligands

The purified nsp16/nsp10 protein complex was labeled with a protein labeling Kit (Monolith, RED-NHS 2nd Generation kit, Cat# MO-L011). In brief, 20 µM of protein was incubated with dye solution (60 µM) in the labeling buffer and the reaction was allowed to proceed at room temperature for 30 min. Each ligand was dissolved in the Microscale thermophoresis (MST) reaction buffer containing 20 mM HEPES pH 7.5, 150 mM NaCl, 0.5% glycerol, and 0.05% Tween 20. Two-fold serial dilutions started from 2 mM ligand concentrations were made in 12 steps. The labeled protein at a final concentration of 20 nM was equally mixed into each ligand reaction (ligand concentration range 500 nM–1 mM). The final reaction mixtures were loaded into premium capillary chips (Monolith Cat# MO-AK005) and measured on a Monolith NT.115 instrument (NanoTemper Technologies) at 20% excitation power and 40% MST power at 25 °C. The results shown here are from two independent experiments. Data were fitted by a single-site binding model in GraphPad Prism (GraphPad Software, San Diego, CA).

### Enzyme activity assay

To assess the relative activity of nsp16/nsp10 on RNA with adenosine or guanosine as the initiating nucleotide, increasing concentrations of purified nsp16/nsp10 were allowed to react with 1 µM ^me7^GpppA- or ^me7^GpppG-capped 25 nt RNA in a buffer containing 50 mM Tris-HCl, pH 8.0, 5 mM KCl, 1 mM DTT, 0.2 mM SAM and 1 mM MgCl_2_. The reactions were incubated at 37 °C for 30 min and stopped by heating at 75 °C for 5 min. The reactions were subjected to LC/MS intact mass analysis. Briefly, nucleic acids in the samples were separated using a Thermo DNAPac™ RP Column (2.1 × 50 mm, 4 µm) on a Vanquish Horizon UHPLC System, followed by mass determination using a Thermo Q-Exactive Plus mass spectrometer. The raw data were deconvoluted using Promass HR (Novatia Inc.). The ratio of the deconvoluted mass peak intensity of the reactants and the expected products were used to estimate the percentage of 2′-O methylation. Results from three independent experiments are shown.

The two RNA substrates used in this assay only differ by 1 base (A vs G) at N_1_ base position: ^me7^GpppAUAGAACUUCGUCGAGUACGCUCAA-[6-FAM]

^me7^GpppGUAGAACUUCGUCGAGUACGCUCAA-[6-FAM]

### Reporting summary

Further information on research design is available in the Nature Research Reporting Summary linked to this article.

## Supplementary information

Supplementary Information

Reporting Summary

Description of Additional Supplementary Files

Supplementary Movie 1

## Data Availability

The information about coding sequences of nsp16 (NCBI reference sequence YP_009725311.1) and nsp10 (NCBI reference sequence: YP_0009725306.1) of the Wuhan seafood market pneumonia virus isolate Wuhan-Hu-1 (NC_045512) used in this study is available at NCBI (https://www.ncbi.nlm.nih.gov/nuccore/NC_045512). Files for atomic coordinates and structure factors were deposited in the Protein Data Bank under accession code 6WKS. Source data are provided as a Source Data file. Correspondence and requests for material should be addressed to Y.K.G. (guptay@uthscsa.edu). Source data are provided with this paper.

## Source data

Source Data
