# Supplementary material for: Structural basis of RNA cap modification by SARS-CoV-2
Source: Nat Commun. 2020 Jul 24;11:3718. doi: 10.1038/s41467-020-17496-8 (PMC7381649; doi:10.1038/s41467-020-17496-8)
Supplement: Supplementary file 1 — Supplementary Information [file 41467_2020_17496_MOESM1_ESM.pdf]

SUPPLEMENTARY INFORMATION

Structural Basis of RNA Cap Modification by SARS-CoV-2 Coronavirus

Thiruselvam Viswanathan[1,2,#], Shailee Arya[1,2,#], Siu-Hong Chan[3], Shan Qi[1,2], Nan Dai[3], Anurag Misra[1,2], Jun-Gyu Park[4], Fatai Oladunni[4], Dmytro Kovalskyy[1], Robert A. Hromas[5], Luis Martinez-Sobrido[4], Yogesh K. Gupta[1,2,6*]

[1] Greehey Children's Cancer Research Institute
[2] Department of Biochemistry and Structural Biology
University of Texas Health San Antonio
8403 Floyd Curl Drive, San Antonio, TX 78229, USA

[3] New England Biolabs, Ipswich, MA

[4] Texas Biomedical Research Institute, San Antonio, TX

[4] Division of Hematology and Oncology, Department of Medicine
University of Texas Health San Antonio
8403 Floyd Curl Drive, San Antonio, TX 78229, USA

[6] Lead contact

[*]Corresponding author:

Y.K.G email: guptay@uthscsa.edu

phone: +1-210-562-9064

[#] These authors contributed equally

SUPLLEMENTARY FIGURES

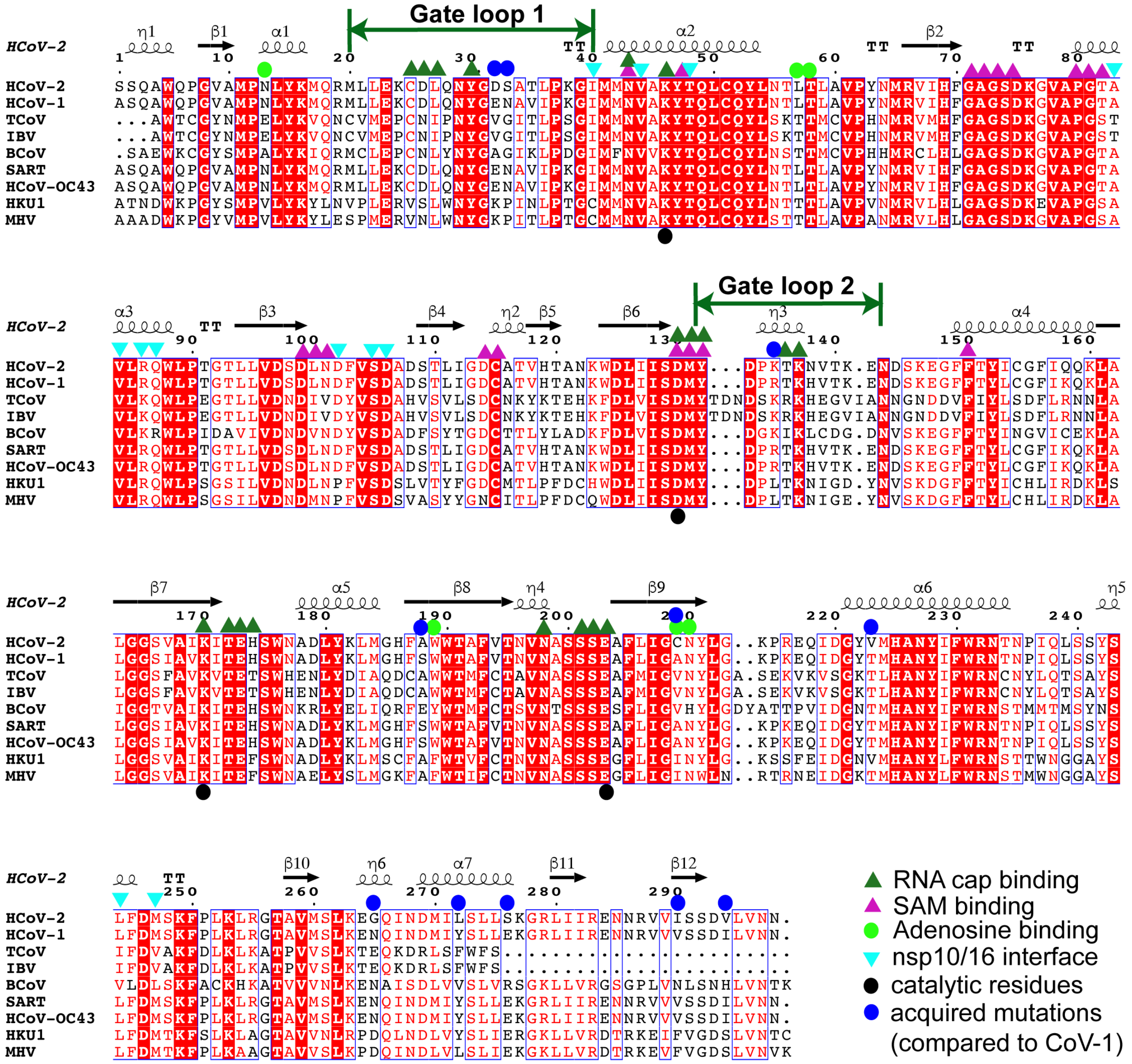

Supplementary Figure 1. Structural elements of nsp16.

The SARS-CoV-2 nsp16 adopts a SAM-MT fold[1]. The secondary structural elements are shown above the aligned protein sequences of nsp16 from nine coronavirus members (HCoV-2, SARS-CoV-2, YP_009725311.1; HCoV-1, SARS-CoV-1, Uniprot ID: P0C6X7; TCoV, turkey coronavirus, YP_001941189.1; IBV, infectious bronchitis virus; BCoV, bat coronavirus,

YP_008439226.1; SART, human SARS CoV, NP_828873.2; HCoV-OC43, human coronavirus OC43; HKU1, human coronavirus, YP_460023.1; and MHV, murine hepatitis virus, YP_209243.1). The protein sequences were first aligned using MUSCLE server (https://www.ebi.ac.uk/Tools/msa/muscle/). The ESPript server[2] was used to assign secondary structural elements of the SARS-CoV-2 nsp16 structure. The substrate (RNA cap) binding regions span two-thirds of the protein, and the regions belonging to the first half participate in SAM binding. The major contributions to cap binding come from gate loop regions 1 and 2. Different symbols atop residues represent their respective involvement in substrate (green triangles), co-factor (magenta triangles), adenosine (bright green circles), and inter-subunit interactions (inverted triangles in cyan). Catalytic residues are annotated by black circles on the bottom. Positions of acquired mutations in SARS-CoV-2 (compared to SARS-CoV-1) are denoted by blue circles.

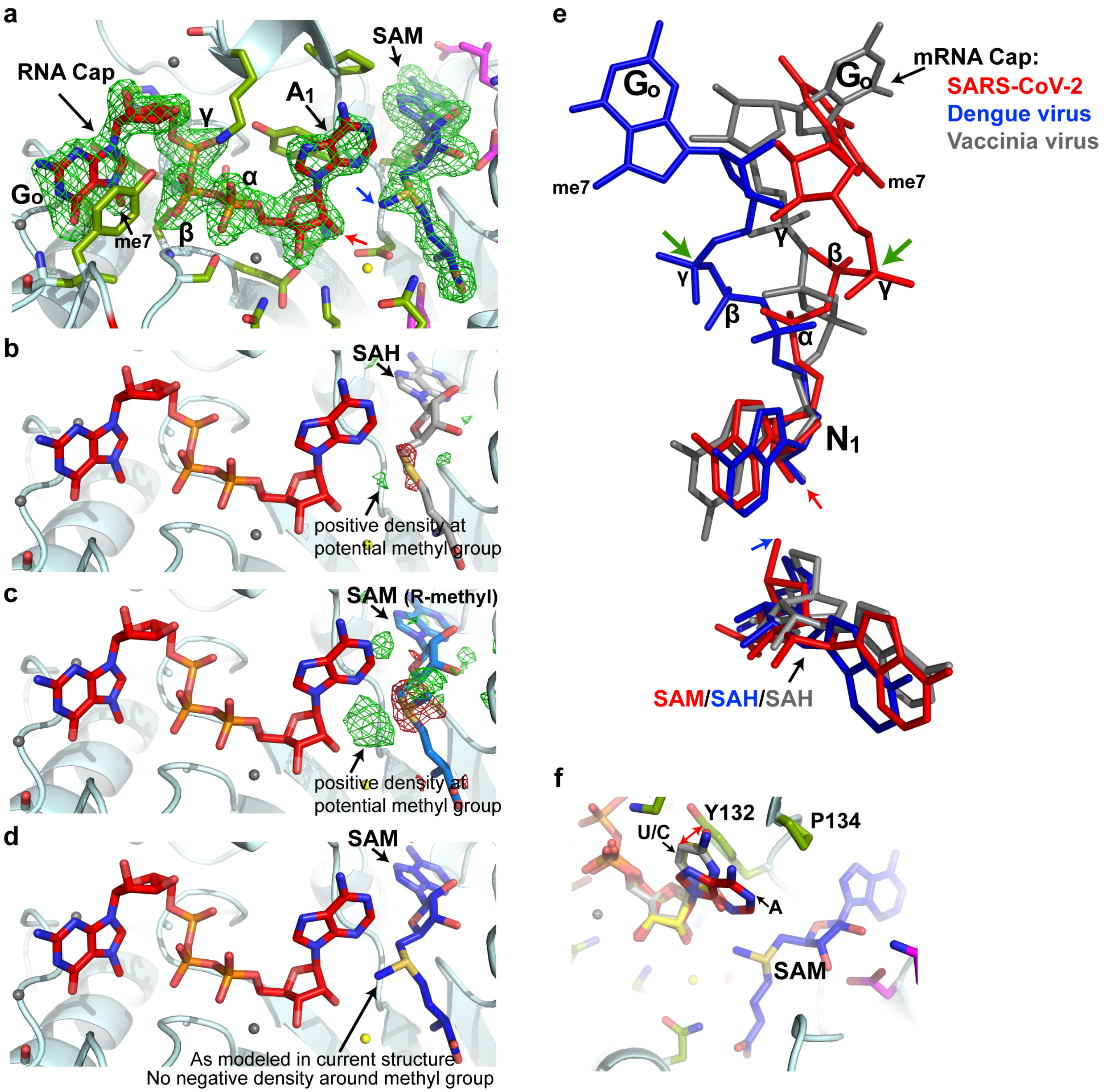

Supplementary Figure 2. Electron density maps of RNA substrate, SAM, and orientation of RNA caps in different viruses.

a) A close-up view of the binding pockets for substrate (RNA cap, red stick) and cofactor (S-adenosyl methionine or SAM, blue stick) analogues. The Fo-Fc electron density omit map for these ligands contoured at 2.8σ is shown as a green mesh. To minimize the possibility of ligand

introduced bias, ligands were excluded from refinement and phase calculations. Positions of the methyl group (in donor SAM), and the acceptor moiety (2'-O in ribose of the target nucleotide A_1) are depicted by blue and red arrows, respectively. b) An Fo-Fc map (contoured at 2.8σ) of the refined model with SAH (grey stick) in the catalytic pocket shows positive (green mesh) density indicative of potential location of methyl group. c) An Fo-Fc map (contoured at 2.8σ) of the refined model with SAM (with methyl group modeled in reverse direction relative to 2'OH) shows strong positive density indicative of the position of methyl group towards the target 2'-OH whereas strong negative density (red mesh) suggests against reverse positioning of the methyl group. These maps, along with the unbiased Fo-Fc omit map (in top panel) helped us to model the methyl group correctly. As modeled in the current structure, there is no negative or positive densities was observed around SAM (d). e) A superposition of N_1 ribose of RNA caps of SARS-CoV-2 (present work, red), Dengue virus (PDB ID: 5DTO, blue), and vaccinia virus (PDB ID: 1AV6, grey) shows 180º rotation in γ-phosphates (green arrows) of RNA caps of SARS-CoV-2 and Dengue viruses. F) A red arrow shows steric clash between Tyr132 side chain and the pyrimidine ring of the U/C base (grey/yellow stick) at N_1 position.

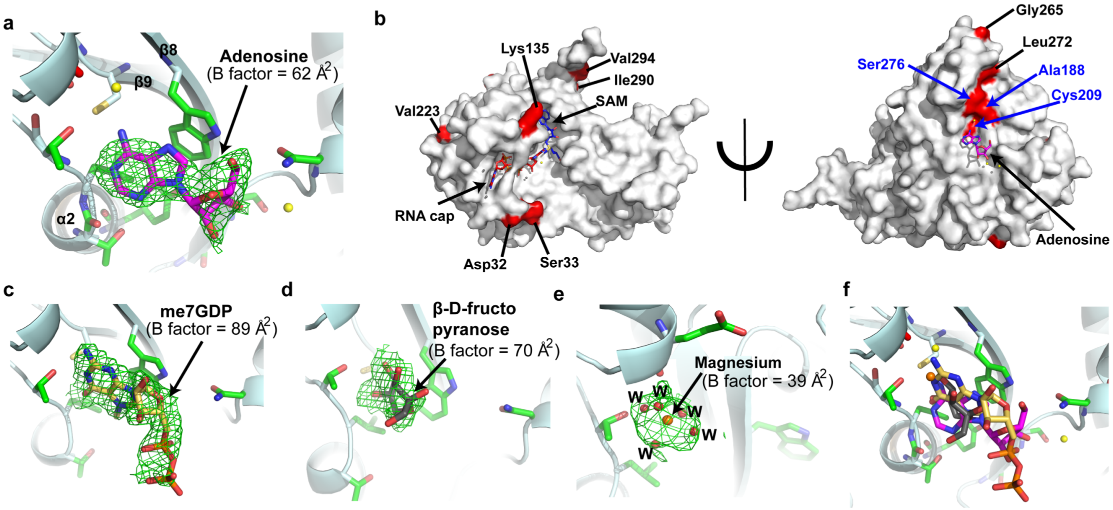

Supplementary Figure 3. Alternative ligand binding site and mapping of acquired mutations in SARS-CoV-2 nsp16.

a) A close-up view of the binding pocket for adenosine (magenta stick). The Fo-Fc electron density omit map for adenosine contoured at 2.8σ is shown as a green mesh. To minimize the possibility of ligand introduced bias, adenosine was excluded from refinement and phase calculations. b) Positions of acquired mutations in SARS-CoV-2 (red surface) compared to from SARS-CoV-1 are mapped on nsp16 surface (light grey). The putative allosteric triad (Ala188, Cys209, Ser276) residues specific to SARS-CoV-2 nsp16 are labeled and marked in blue. c-f) A variety of ligands such as ^{me7}GDP (beige, PDB ID: 6WQ3), β-D-fructopyranose (grey stick, PDB ID: 6W4H) can occupy this alternative site in SARS-CoV-2, and Mg^{2+} (orange sphere surrounded by water molecules, PDBID: 2XYR) in SARS-CoV-1 nsp16.

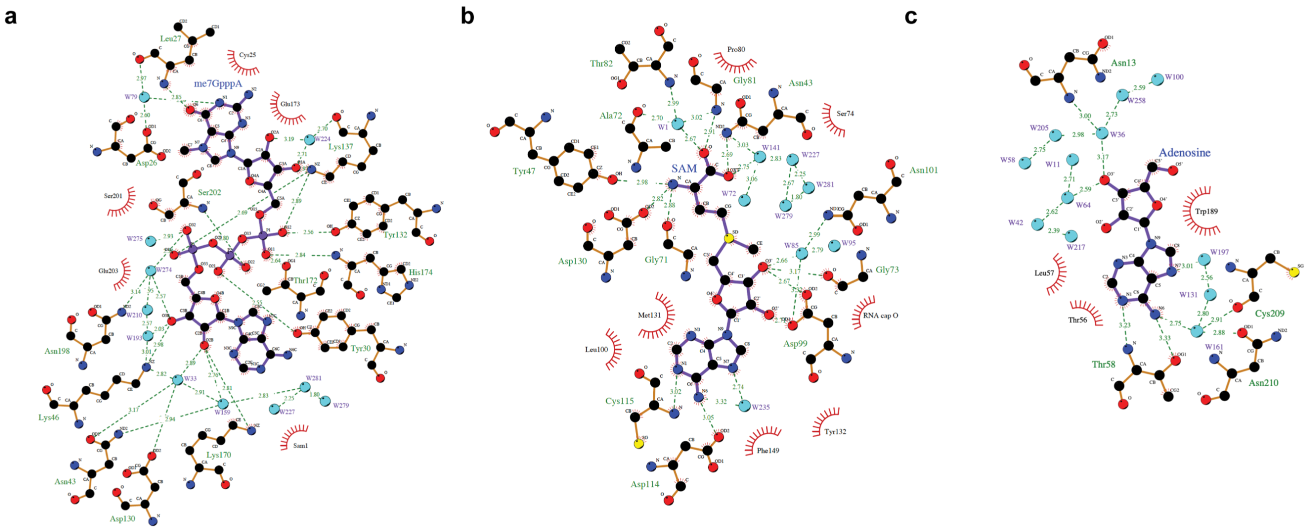

Supplementary Figure 4. Protein-ligand interaction network.

Network of Nsp16/nsp10 interaction with RNA cap analogue me7GpppA (a), S-adenosyl methionine or SAM (b), and adenosine (c). The green dashed lines represent hydrogen bonding and the W represents a water molecule. These figures were generated using LigPlot+ program[3].

SUPLLEMENTARY TABLES

Supplementary Table 1. Data collection and refinement statistics (molecular replacement)

	nsp16/nsp10/SAM/$^{me7}G_opppA$
**Data collection**	
Space Group	$P3_121$
Cell dimensions	
a=b, c (Å)	168, 52.3
α=β, γ (°)	90, 120
Resolution (Å)	1.8
R_{sym} (%)	13.71 (165) *
$I/\sigma I$	14.15 (1.35)
Completeness (%)	99.9 (100)
Redundancy	20.8 (20.2)
**Refinement**	
Resolution (Å)	30-1.8
Number of reflections	74492 (5390)
R_{work} (%) / R_{free} (%)	14.3 / 18.1
Nonhydrogen atoms	
Protein	3526
Ligands	114
Water	252
Average B-factors (Å^2)	
Protein / Catalytic tetrad (K46, D130, K170, E203)	42.9 / (27.5, 29.7, 29.5, 28.3)
Ligands (SAM, RNA cap, adenosine)/	43.1 (40, 48, 62) /
Water	47.8
R.m,s, deviations	
Bond lengths (Å)	0.014
Bond angles (°)	1.85

*Values for outermost shell are given in parentheses.

SUPLLEMENTARY REFERENCES

[1] Martin, J. L., and McMillan, F. M. (2002) SAM (dependent) I AM: the S-adenosylmethionine-dependent methyltransferase fold, *Curr Opin Struct Biol 12*, 783-793.
[2] Robert, X., and Gouet, P. (2014) Deciphering key features in protein structures with the new ENDscript server, *Nucleic Acids Res 42*, W320-324.
[3] Laskowski R. A., Swindells M. B. (2011). LigPlot+: multiple ligand-protein interaction diagrams for drug discovery. *J. Chem. Inf. Model.*, **51**, 2778-2786.
