# Supplementary material for: Structural basis of RNA cap modification by SARS-CoV-2
Source: Nat Commun. 2020 Jul 24;11:3718. doi: 10.1038/s41467-020-17496-8 (PMC7381649; doi:10.1038/s41467-020-17496-8)
Supplement: Supplementary file 2 — Reporting Summary [file 41467_2020_17496_MOESM2_ESM.pdf]

nature research

Corresponding author(s): Yogesh K. Gupta

Last updated by author(s): Jun 29, 2020

Reporting Summary

Nature Research wishes to improve the reproducibility of the work that we publish. This form provides structure for consistency and transparency in reporting. For further information on Nature Research policies, see our Editorial Policies and the Editorial Policy Checklist.

Statistics

For all statistical analyses, confirm that the following items are present in the figure legend, table legend, main text, or Methods section.

n/a	Confirmed	
[x]	[ ]	The exact sample size (*n*) for each experimental group/condition, given as a discrete number and unit of measurement
[ ]	[x]	A statement on whether measurements were taken from distinct samples or whether the same sample was measured repeatedly
[x]	[ ]	The statistical test(s) used AND whether they are one- or two-sided<br>*Only common tests should be described solely by name; describe more complex techniques in the Methods section.*
[x]	[ ]	A description of all covariates tested
[x]	[ ]	A description of any assumptions or corrections, such as tests of normality and adjustment for multiple comparisons
[x]	[ ]	A full description of the statistical parameters including central tendency (e.g. means) or other basic estimates (e.g. regression coefficient) AND variation (e.g. standard deviation) or associated estimates of uncertainty (e.g. confidence intervals)
[x]	[ ]	For null hypothesis testing, the test statistic (e.g. *F*, *t*, *r*) with confidence intervals, effect sizes, degrees of freedom and *P* value noted<br>*Give P values as exact values whenever suitable.*
[x]	[ ]	For Bayesian analysis, information on the choice of priors and Markov chain Monte Carlo settings
[x]	[ ]	For hierarchical and complex designs, identification of the appropriate level for tests and full reporting of outcomes
[x]	[ ]	Estimates of effect sizes (e.g. Cohen's *d*, Pearson's *r*), indicating how they were calculated

Our web collection on statistics for biologists contains articles on many of the points above.

Software and code

Policy information about availability of computer code

Data collection	NECAT-24 ID C beamline of Advanced Photon Source, Argonne National Laboratory, Chicago, IL, USA was used for X-ray data collection. NGC Discovery FPLC (BioRad) was used for protein purification. The MST (microscale thermophoresis) data to probe protein-ligand interactions was measured using Monolith NT (Nanotemper). The methyltransferase assays were done using Vanquish Horizon UHPLC System and Thermo Q-Exactive Plus mass spectrometer.
Data analysis	The X-ray diffraction data was processed using RAPD data processing pipeline at NECAT-24ID. The RAPD used combination of XDS, AIMLESS, POINTLESS, programs. Grade (Global Phasing Ltd.) was used for generating ligand restraints. XDS (v 2019), aimless, truncate, freeflag, mtz2various, PHASER programs integrated in CCP4 (v 7.0) were used in X-ray diffraction data processing. REFMAC (v5) was used for refinement. Latest versions of MUSCLE and ESpript programs (references provided in the supplementary information) were used for structure-based protein alignment. Coot (v 0.8) was used for model building. Pymol (Version 2.0 Schrödinger LLC.) was used for illustration of structure figures. We used GraphPad Prism software (version 8.0) for analyzing the ligand binding affinities. Adobe illustrator was used for final figure generation.

For manuscripts utilizing custom algorithms or software that are central to the research but not yet described in published literature, software must be made available to editors and reviewers. We strongly encourage code deposition in a community repository (e.g. GitHub). See the Nature Research guidelines for submitting code & software for further information.

Data

Policy information about availability of data

All manuscripts must include a data availability statement. This statement should provide the following information, where applicable:

- Accession codes, unique identifiers, or web links for publicly available datasets
- A list of figures that have associated raw data
- A description of any restrictions on data availability

Files for atomic coordinates and structure factors were deposited in the Protein Data Bank under accession codes 6WKS.
The coding sequences of nsp16 (NCBI reference sequence YP_009725311.1) and nsp10 (NCBI reference sequence: YP_0009725306.1) of the Wuhan seafood market pneumonia virus isolate Wuhan-Hu-1 (NC_045512) were used in this study. The sequence information is available at NCBI (https://www.ncbi.nlm.nih.gov/nuccore/NC_045512). Correspondence and requests for material should be addressed to Y.K.G. (guptay@uthscsa.edu).

Field-specific reporting

Please select the one below that is the best fit for your research. If you are not sure, read the appropriate sections before making your selection.

[x] Life sciences [ ] Behavioural & social sciences [ ] Ecological, evolutionary & environmental sciences

For a reference copy of the document with all sections, see nature.com/documents/nr-reporting-summary-flat.pdf

Life sciences study design

All studies must disclose on these points even when the disclosure is negative.

Sample size	Not Applicable
Data exclusions	No data were excluded
Replication	Biochemical experiments were replicated three times independently. The other experiments (crystallographic studies) were not replicated, which is standard in the X-ray crystallography field.
Randomization	Not Applicable. Randomization is not possible in X-ray crystallographic studies as it is essential to have the full information about the material (protein and ligands) used in order to determine 3-dimensional structures.
Blinding	Not Applicable. Blinding is not required in X-ray crystallographic studies as it is essential to have the full information about the material (protein and ligands) used in the study in order to determine 3-dimensional structures.

Reporting for specific materials, systems and methods

We require information from authors about some types of materials, experimental systems and methods used in many studies. Here, indicate whether each material, system or method listed is relevant to your study. If you are not sure if a list item applies to your research, read the appropriate section before selecting a response.

Materials & experimental systems

n/a	Involved in the study	
[x]	[ ]	Antibodies
[x]	[ ]	Eukaryotic cell lines
[x]	[ ]	Palaeontology and archaeology
[x]	[ ]	Animals and other organisms
[x]	[ ]	Human research participants
[x]	[ ]	Clinical data
[x]	[ ]	Dual use research of concern

Methods

n/a	Involved in the study	
[x]	[ ]	ChIP-seq
[x]	[ ]	Flow cytometry
[x]	[ ]	MRI-based neuroimaging
