# Supplementary material for: Structural basis of RNA cap modification by SARS-CoV-2
Source: Nat Commun. 2020 Jul 24;11:3718. doi: 10.1038/s41467-020-17496-8 (PMC7381649; doi:10.1038/s41467-020-17496-8)
Supplement: Supplementary file 3 — Description of Additional Supplementary Files [file 41467_2020_17496_MOESM3_ESM.pdf]

Description of Additional Supplementary Files

File Name: Supplementary Movie 1
Description: An animation showing conformational changes in nsp16 of the nsp16/nsp10 ternary complex (present work) as the enzyme transitions from the binary (S-adenosyl methionine or SAM-bound) state. Light cyan cartoons, nsp16; green sticks, residues of nsp16 engaged in cap (red stick) and SAM (not shown) binding. The SAM-bound SARS-CoV nsp16/nsp10 (PDB ID: 3R24) structure was used as the binary state in this calculation.
